# Pharmacological switch in Aβ-fiber stimulation-induced spinal transmission in mice with partial sciatic nerve injury

**DOI:** 10.1186/1744-8069-4-25

**Published:** 2008-07-11

**Authors:** Misaki Matsumoto, Weijiao Xie, Lin Ma, Hiroshi Ueda

**Affiliations:** 1 Division of Molecular Pharmacology and Neuroscience, Nagasaki University Graduate School of Biomedical Sciences, Nagasaki 852-8521, Japan

## Abstract

**Background:**

We have previously demonstrated that different spinal transmissions are involved in the nociceptive behavior caused by electrical stimulation of Aβ-, Aδ- or C-fibers using a Neurometer^® ^in naïve mice. In this study, we attempted to pharmacologically characterize the alteration in spinal transmission induced by partial sciatic nerve injury in terms of nociceptive behavior and phosphorylation of extracellular signal-regulated kinase (pERK) in the spinal dorsal horn.

**Results:**

Aβ-fiber responses (2000-Hz), which were selectively blocked by the AMPA/kainate antagonist CNQX in naïve mice, were hypersensitized but blocked by the NMDA receptor antagonists MK-801 and AP-5 in injured mice in an electrical stimulation-induced paw withdrawal (EPW) test. Although Aδ-fiber responses (250-Hz) were also hypersensitized by nerve injury, there was no change in the pharmacological characteristics of Aδ-fiber responses through NMDA receptors. On the contrary, C-fiber responses (5-Hz) were hyposensitized by nerve injury. Moreover, Aδ- and C-, but not Aβ-fiber stimulations significantly increased the number of pERK-positive neurons in the superficial spinal dorsal horns of naïve mice, and corresponding antagonists used in the EPW test inhibited this increase. In mice with nerve injury, Aβ- as well as Aδ-fiber stimulations significantly increased the number of pERK-positive neurons in the superficial spinal dorsal horn, whereas C-fiber stimulation decreased this number. The nerve injury-specific pERK increase induced by Aβ-stimulation was inhibited by MK-801 and AP-5, but not by CNQX. However, Aβ- and Aδ-stimulations did not affect the number or size of pERK-positive neurons in the dorsal root ganglion, whereas C-fiber-stimulation selectively decreased the number of pERK-positive neurons.

**Conclusion:**

These results suggest that Aβ-fiber perception is newly transmitted to spinal neurons, which originally receive only Aδ- and C-fiber-mediated pain transmission, through NMDA receptor-mediated mechanisms, in animals with nerve injury. This pharmacological switch in Aβ-fiber spinal transmission could be a mechanism underlying neuropathic allodynia.

## Introduction

Primary afferent fibers have been classified into three major types, unmyelinated C-, thinly myelinated Aδ-, and myelinated Aβ-fibers. The nociceptors of C-fiber and Aδ-fiber primary afferent neurons transduce noxious chemical, mechanical or thermal stimuli into depolarizing potentials, which in turn cause nociceptive responses. On the other hand, the stimulation of Aβ-fibers is mostly thought to induce an innocuous tactile sensation. We recently characterized the nociceptive responses through these three different types of fiber activated by different frequencies of electrical stimuli using a Neurometer^® ^(electrical stimulation-induced paw flexion test; EPF test) [1]. In such studies, Aβ-fiber responses were unique, since an AMPA/kainate receptor antagonist selectively blocked them, while NK1 and/or NMDA receptor antagonists blocked C- and Aδ-fiber responses. Thus, pharmacological characterization of these responses may contribute to our understanding of pain transmission.

On the other hand, neuropathic pain has attracted the concern of many neuroscientists in terms of the mechanisms underlying spinal neurotransmission. Neuropathic pain results in two characteristic abnormal nociceptive behaviors, namely, hyperalgesia and allodynia. Of particular interest are the mechanisms of allodynia, which include the conversion of innocuous stimuli to pain. We previously found that both Aδ-fiber- and Aβ-fiber-mediated responses of mice with partial sciatic nerve injury were hypersensitized in the EPF test [1], although a pharmacological characterization of such hypersensitivity in terms of spinal neurotransmission has not been performed.

Extracellular signal-regulated kinases (ERKs), representing one of the major subfamilies of mitogen-activated protein kinases (MAPKs), are phosphorylated following membrane depolarization and Ca^2+ ^influx [2]. ERKs have been reported to be immediately activated after noxious stimulation in the neurons of the dorsal root ganglions (DRGs) and spinal dorsal horn in a stimulus intensity-dependent manner [3,4]. Therefore, ERK phosphorylation in DRGs and the spinal dorsal horn could be a biochemical marker of activated neurons, allowing us to visualize pain-signaling pathways and obtain more objective evidence of neurotransmission. In the present study, we present behavioral and biochemical evidence for Aβ-fiber-mediated allodynia (hypersensitization) and its associated alteration in synaptic neurotransmission at the level of the spinal cord in animals with neuropathic pain.

## Results

### Determination of spinal pain transmitters using an electrical stimulation-induced paw withdrawal (EPW) test

**Figure 1:**
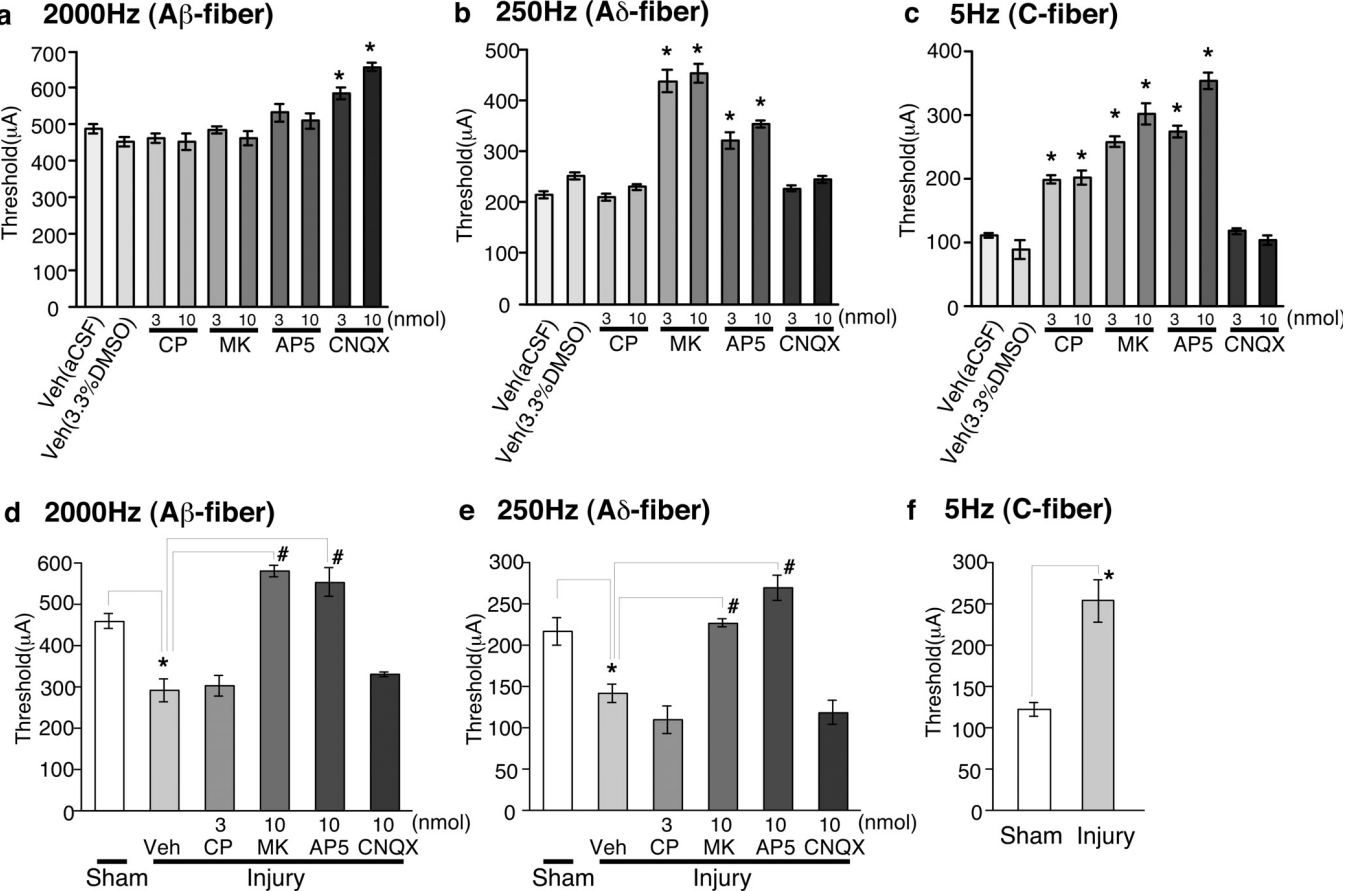
**Pharmacological plasticity in the spinal antagonism of Aβ-, Aδ-, and C-fiber stimulation-mediated nociceptive behaviors in nerve-injured mice.** The threshold represents the minimum intensity (μA) required to produce a paw withdrawal response to electrical stimulation with 2000 Hz (Aβ-fiber) (a, d), 250 Hz (Aδ-fiber) (b, e), and 5 Hz (C-fiber) (c, f). Vehicle or drugs were administrated intrathecally to naïve (a-c) or nerve-injured mice (d-f), 10 min prior to the electrical stimulation. Vehicle (Veh; aCSF or 3.3% DMSO), CP-99994 (CP), MK-801 (MK). *: *p *< 0.05 *vs. *vehicle (aCSF or 3.3% DMSO in a-c) or sham operation (d-f). #: *p *< 0.05 *vs. *injury-vehicle. Data represent the means ± S.E.M. from experiments using at least 6 mice.

Transcutaneous nerve stimuli, specifically three sine-wave pulses with frequencies of 2000, 250 or 5 Hz, to activate Aβ-, Aδ- or C-fibers, respectively [5-7], were applied to the right hind paw of a mouse. The threshold of current (μA) for each stimulus was determined by evaluating paw withdrawal behavior. Each stimulus including 2000 Hz (Aβ-fiber), which described as an unpleasant vibrating perception [8] or light tickling sensation [9], caused the paw withdrawal response in mice. In control (aCSF) mice, the withdrawal thresholds for each frequency were 486.3 ± 12.9 μA (2000 Hz, Aβ-fiber), 212.0 ± 6.5 μA (250 Hz, Aδ-fiber) and 100.8 ± 4.2 μA (5 Hz, C-fiber). To characterize spinal pain transmission through each fiber, representative antagonists, namely, the substance P (NK1) receptor antagonist CP-99994, the competitive and non-competitive NMDA receptor antagonists AP-5 and MK-801, respectively, and the non-selective antagonist of AMPA/kainate (non-NMDA) receptors CNQX, were intrathecally administered 10 min prior to electrical stimulation. Antagonists or vehicle (aCSF or 3.3% DMSO solution) alone induced no change in locomotion behavior, but short-lasting scratching behavior was observed only immediately after the administration of CNQX (10 nmol). In accordance with findings from the EPF test [1], which is the prototype for the electrical stimulation-induced paw withdrawal (EPW) test, the threshold at 2000 Hz was increased only by pretreatment with CNQX (3 and 10 nmol), but not by CP-99994, AP-5 or MK-801 (3 nmol and 10 nmol), as shown in Figure 1a. On the other hand, the threshold at 250 Hz was increased by both AP-5 and MK-801, but not by CP-99994 or CNQX, while the threshold at 5 Hz was increased by CP-99994, AP-5 and MK-801, but not by CNQX (Figure 1b and 1c). These results are also consistent with the previous findings in the EPF test [1].

### Altered spinal pain transmission through Aβ-fibers in nerve-injured mice

The EPW test was performed with mice that had received partial sciatic nerve ligation, according to the method of Malmberg and Basbaum [10]. In the present study we used a dose of each antagonist to produce a maximal effect in naive mice (see Figure 1a–c). As shown in Figure 1d, the threshold at 2000 Hz was significantly decreased in these injured mice. Although the intrathecal injection of CP-99994 (3 nmol, i.t.) at 10 min prior to the EPW test did not affect this injury-induced decrease in the threshold at 2000 Hz, both MK-801 (10 nmol, i.t.) and AP-5 (10 nmol, i.t.) completely reversed this decrease. However, pretreatment with CNQX (10 nmol, i.t.) did not affect the threshold. Similar sensitization was also observed with 250 Hz stimuli, but the spinal antagonism remained unchanged in injured mice (Figure 1e). As previously reported with the EPF test [1], the threshold with 5 Hz stimuli in the EPW test was increased by nerve injury (Figure 1f).

### Distinct spinal neurotransmission is involved stimuli-induced spinal ERK phosphorylation in different fibers

**Figure 2:**
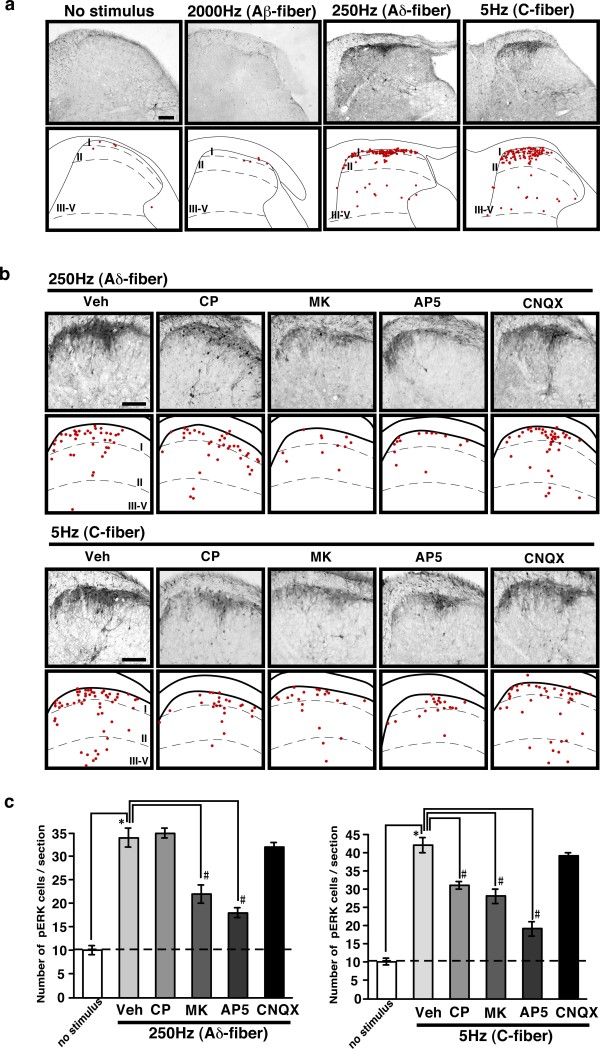
**Pharmacological characterization of the activation of dorsal horn neurons by Aβ-, Aδ- and C-fiber electrical stimulations.** The activation was evaluated by the immunoreactivity for pERK. (a) Representative pictures of pERK signals in the ipsilateral spinal dorsal horn after electrical stimulation to the right hind paw (see Methods). The figures in the bottom of each picture indicate pERK signals, which were used for pERK quantification (see Methods). Representative pictures of spinal antagonism of the pERK signals induced by 250 or 5 Hz stimuli (b), and the results of pERK quantification (c).: vehicle (Veh; aCSF), CP-99994 (CP) (10 nmol), MK-801 (MK) (30 nmol), AP-5 (30 nmol), CNQX (10 nmol). *: *p *< 0.05 *vs. *no stimulus, #: *p *< 0.05 *vs. *vehicle. Data represent the means ± S.E.M. from experiments using at least 4 mice. Scale bar = 100 μm.

ERK has been reported to be immediately phosphorylated after noxious stimulation in the neurons of dorsal root ganglions (DRGs) and the spinal dorsal horn in a stimulus intensity-dependent manner [3,4]. Therefore, we used phosphorylated ERK (pERK) as a biochemical marker of activated neurons to visualize pain-signaling pathways. The higher current intensities (2000 Hz, 1000 μA; 250 Hz, 2000 μA; 5 Hz, 2000 μA; 1 min) of electrical stimulation was used to evaluate pERK signals, since a relatively high-intensity noxious stimulation is required for ERK activation [3,4]. As expected, no significant pERK signal was observed by stimulation of the threshold at which paw withdrawal response was induced in the EPW test (Data not shown). We took special care to select the current intensity (1000 μA) for Aβ-fiber stimulation, since Aβ specificity is only observed below 1300 μA of 2000 Hz stimulation in electrophysiological studies involving intracellular recordings from DRG neurons [6]. However, the 2000-Hz stimulation caused no significant pERK signal in the spinal dorsal horn, although 250- and 5-Hz stimuli produced significant signals, mainly in the superficial spinal dorsal horn (lamina I–II), as shown in Figure 2a. The pERK signals induced by 250-Hz stimuli were more concentrated in the outer layer of the dorsal horn (lamina I), while 5 Hz-induced signals were found in slightly broader (lamina I and II) regions. In addition to these signals in spinal neurons, there were significant diffuse signals induced in the superficial dorsal horn by 250 and 5 Hz stimuli, but not by 2000 Hz stimuli. These seem to be due to the activation of central terminals of primary fibers and dendrites of spinal neurons. Some pERK-positive cells were also observed in deeper regions of the dorsal horn (lamina III–V), induced by both 250 and 5 Hz stimuli. No pERK signal was observed without anti-pERK antibodies (data not shown).

In order to characterize spinal antagonisms in terms of nerve fiber-specific ERK activation, we used the following antagonists: CP-99994 (10 nmol, i.t.), MK-801 (30 nmol, i.t.), AP-5 (30 nmol, i.t.) and CNQX (10 nmol, i.t.). None of these antagonist treatments produced specific pERK signals. As shown in Figure 2b and 2c, the 250 Hz stimuli-induced pERK signals in spinal neurons were significantly inhibited by MK-801 or AP-5, but not by CP-99994 or CNQX. However, these antagonists did not completely inhibit the diffuse signals, and this is possibly why the substantial activation of primary afferent central terminals remains unchanged. Similarly the 5 Hz stimuli-induced signals in neurons were significantly inhibited by CP-99994, MK-801 and AP-5, but not by CNQX, while the diffuse signals partially remained.

### Altered Aβ-fiber-induced spinal ERK phosphorylation in nerve-injured mice

**Figure 3:**
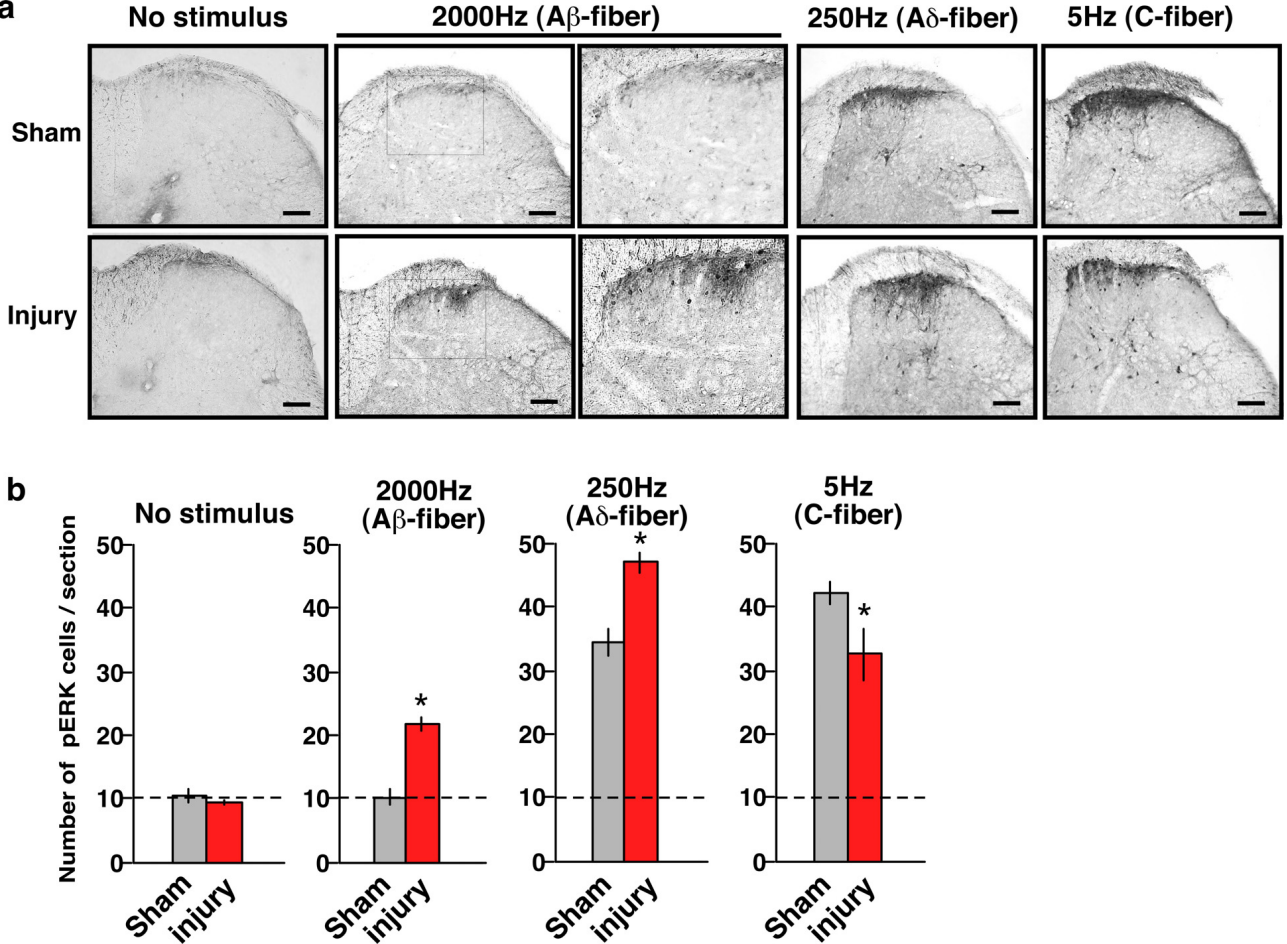
**Altered pERK signals in the spinal dorsal horn induced by Aβ-, Aδ-, and C-fiber electrical stimulation in nerve-injured mice.** (a) Representative pictures of pERK signals in the ipsilateral spinal dorsal horn after electrical stimulation to the right hind paw. (b) Results of pERK quantification in the spinal dorsal horn. *: *p *< 0.05 *vs. *sham. Data represent the means ± S.E.M. from experiments using at least 6 mice. Scale bar = 100 μm.

Sham operation or sciatic nerve injury alone induced no specific pERK signals in spinal dorsal horn neurons 7 days later, as shown in Figure 3a, b. In sham-operated mice, the 2000-Hz stimulation induced no significant pERK signal in the spinal dorsal horn, similar to the result in naïve mice (Figure 3a, b). By contrast, the significant and specific 2000-Hz stimuli-induced signals were newly observed in neurons in the superficial dorsal horns (lamina I–II) of nerve-injured mice, but not in neurons in the deeper regions of the dorsal horn (lamina III–V). On the other hand, the number of pERK-positive spinal neurons observed after 250 Hz stimuli was increased in injured mice, while the number of 5 Hz stimuli-induced pERK-positive spinal neurons was decreased (Figure 3a, b).

**Figure 4:**
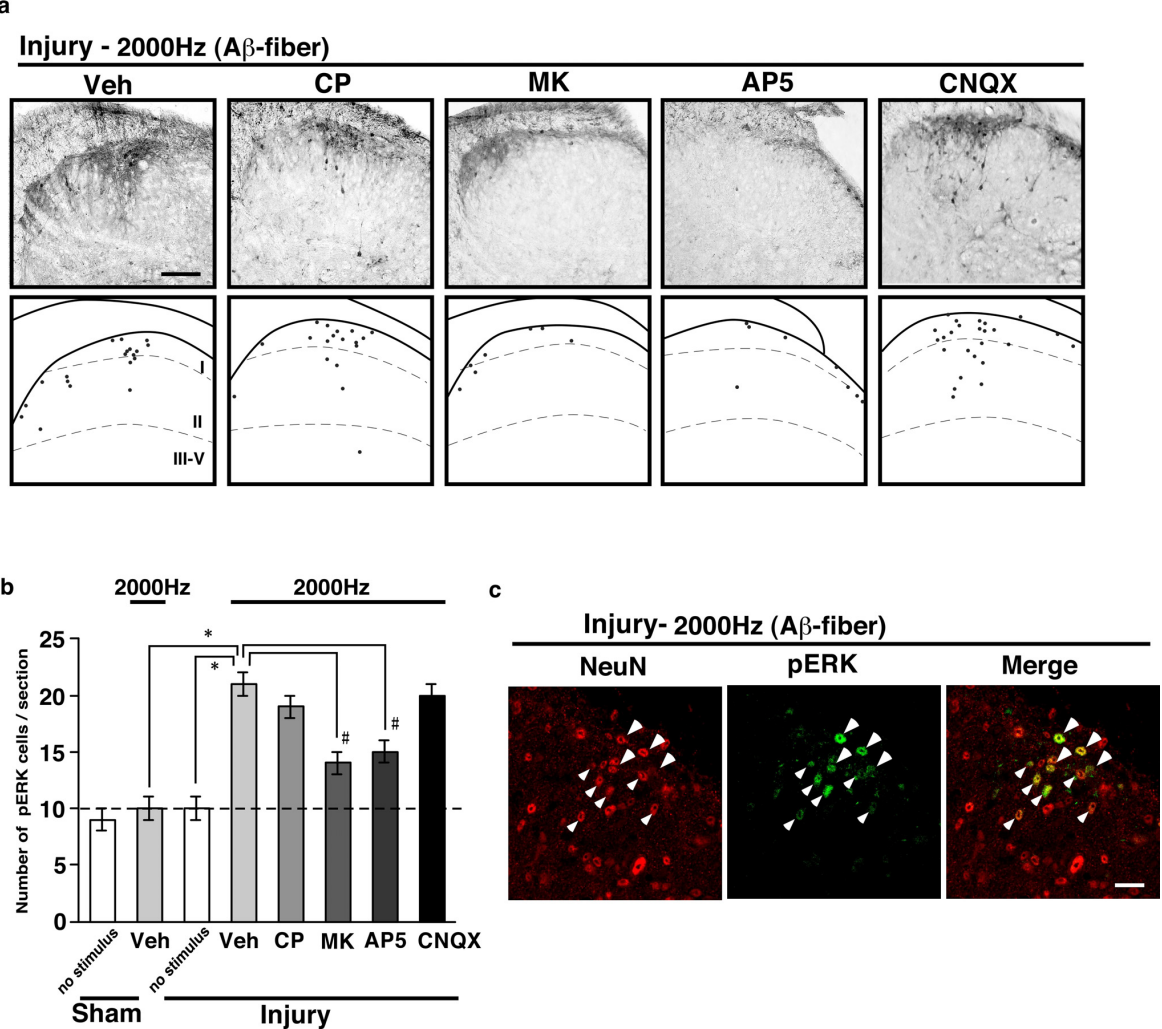
**Altered spinal antagonisms of pERK signals induced by Aβ-fiber stimuli in nerve-injured mice. **Representative pictures of spinal antagonisms of pERK signals induced by 2000 Hz stimuli (a) and the results of pERK quantification (b).: vehicle (Veh; aCSF), CP-99994 (CP) (10 nmol), MK-801 (MK) (30 nmol), AP-5 (30 nmol), CNQX (10 nmol). *: *p *< 0.05 *vs. *sham-vehicle or injury-no stimulus. #: *p *< 0.05 *vs. *injury-vehicle. Data represent the means ± S.E.M. from experiments using at least 4 mice. (c) Double-immunostaining images for pERK (green) and the neural marker NeuN (red) after Aβ-fiber stimuli in the spinal cords of nerve-injured mice. Scale bar = 100 μm for (a) and 20 μm for (c).

In addition, we further examined the spinal antagonism of Aβ-fiber-mediated pERK signals newly observed in nerve-injured mice. These signals were significantly inhibited by MK-801 (30 nmol) or AP-5 (30 nmol), but not by CP-99994 (10 nmol) or CNQX (10 nmol), which had been intrathecally administered 10 min prior to electrical stimulation (Figure 4a, b). All of these pERK-positive signals in dorsal horn neurons in injured mice were colocalized with NeuN signals, suggesting that they can be attributed to the activation of spinal neurons (Figure 4c).

### The specificity of electrical stimulation in terms of ERK phosphorylation in DRG neurons.

**Figure 5:**
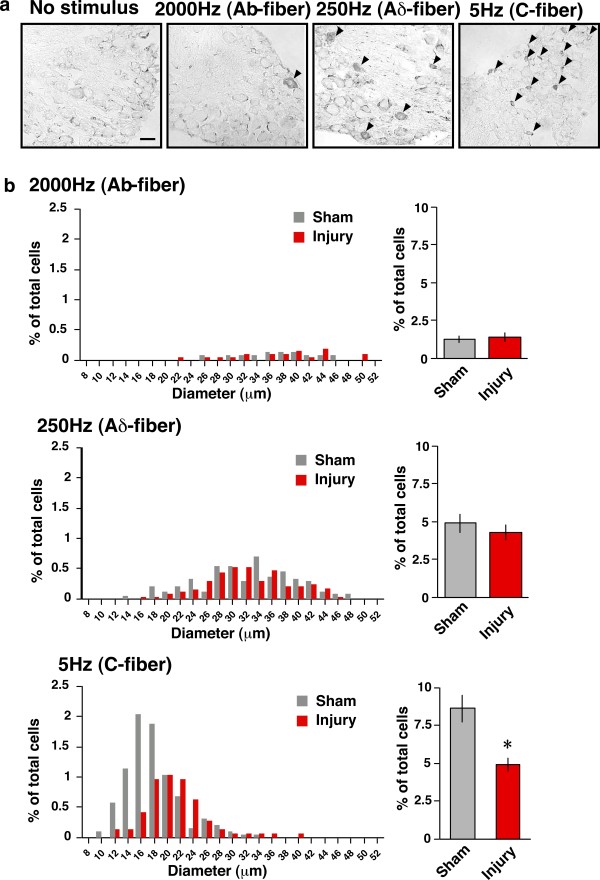
**The specificity of electrical stimulation in terms of ERK phosphorylation in the DRG.** (a) Representative pictures of pERK in the ipsilateral DRG after electrical stimulation to the right hind paw. Arrowheads indicate pERK-positive DRG neurons. (b) The size distribution of pERK-positive DRG cells (left panel) and its relative comparison to the total number of cells (right panel) in sham-operated and nerve-injured mice. *: *p *< 0.05 *vs. *sham. Data represent the means ± S.E.M. from experiments using at least 4 mice. Scale bar = 50 μm.

 As 2000-Hz stimuli caused an unexpected activation of spinal neurons in nerve-injured mice, we further examined the specific activation of dorsal root ganglion (DRG) neurons in sham-operated and injured mice. Control treatment (Figure 5a) and sciatic nerve injury alone (data not shown) did not induce specific pERK signals in the DRG. As shown in Figure 5a, b, the 2000 Hz stimuli-induced pERK positive signals were similarly distributed in medium/large-sized DRG neurons compared with 250 Hz-induced ones (>24 μm), but there were fewer pERK-positive neurons in the former preparation than in the latter one (2000 Hz: 1.16 ± 0.23%, 250 Hz: 4.82 ± 0.60%). The distribution of pERK-positive DRG neurons following 5 Hz stimulation was distinctly restricted to small-diameter cells (<24 μm) and the number of pERK-positive neurons was more abundant (Figure 5a, b). In nerve-injured mice, there was no change in the distribution profile or the number of pERK-positive neurons induced by 2000 and 250 Hz stimuli. However, the smaller DRG neurons (<18 μm) in nerve-injured mice lost pERK signals following stimulation at 5 Hz.

## Discussion

We recently developed a novel test to classify sensory fibers in terms of their EPF responses to different frequencies of electrical stimuli using a Neurometer^® ^[1]. This Neurometer^® ^device has been validated by many clinical studies as a useful tool in the clinical evaluation and management of neurologic disorders [11-13]. Wallace et al. [9] reported that the patient described the 2000 Hz stimuli as a "light tickle", the 250 Hz stimuli as a "prickly feeling" and the 5 Hz stimuli as a deep warmth or coolness. Therefore, the flexor responses (EPF test) or the withdrawal responses (EPW test) observed in mice seem to be the result of escape from the unpleasant (but not nociceptive) 2000 Hz stimuli, or the nociceptive 250 and 5 Hz stimuli. Recently, Koga et al. [6] reported that these three different frequencies of electrical stimuli applied with a Neurometer^® ^selectively activate Aβ-, Aδ- or C-fibers in an electrophysiological study using rats. The fiber-specificity of the Neurometer^® ^was supported by the present study, in which spinal transmission was pharmacologically characterized in terms of nociceptive behavior and phosphorylation of extracellular signal-regulated kinase (pERK) in the spinal dorsal horn. The 250 Hz (Aδ-fiber) stimuli induced pERK signals in medium/large-sized DRG neurons and in neurons of the lamina I layer of the dorsal horn. The intrathecal treatments with NMDA receptor antagonists largely inhibited both the pERK signals in the dorsal horn and nociceptive behavior. The 5 Hz (C-fiber) stimuli induced pERK signals in small-sized DRG neurons and in lamina I and II neurons. Both NK1 receptor antagonist and NMDA receptor antagonists blocked spinal pERK signals and nociceptive behavior. On the other hand, the 2000 Hz (Aβ-fiber) stimuli also induced pERK signals in less abundant medium/large-sized DRG neurons, but not in the spinal dorsal horn. The lack of pERK signals induced by 2000 Hz stimuli was consistent with the report by Ji et al. [3], who demonstrated that no pERK signals were observed in the dorsal horn following Aβ-fiber stimulation, which was verified electrophysiologically. This finding is contrast with our behavioral study, in which 2000-Hz stimuli caused significant paw withdrawal responses that were blocked by intrathecally administered CNQX, an AMPA/kainate antagonist. From the report that rapid pERK signals are caused by an increase in cellular Ca^2+ ^concentration [2,14], the lack of pERK signals in spinal neurons after Aβ-fiber stimulation might be explained by the fact that AMPA/kainate receptors have lower Ca^2+ ^permeability than NMDA receptors [15]. AMPA/kainate receptor antagonist-sensitive Aβ-fiber-mediated neurotransmission is also supported by previous reports [16,17]. Although there is an inconsistency in the current intensities and in the duration of electrical stimulation between the behavioral EPW test and the pERK experiment, the fiber-specificity seems to be conformed, since the pharmacological characterization of C-(5 Hz) and Aδ-fiber stimuli (250 Hz)-induced pERK signals is consistent with the behavioral studies in the present study. Furthermore, as Koga et al. [6] reported that Aβ-specificity is retained in DRG electrophysiological studies as far as the current intensity of 2000 Hz below 1300 μA is used, the Aβ-fiber specificity by 2000 Hz, 1000 μA stimuli in the present study also seems to be retained, though this stimulation failed to induce ERK activation in naïve mice. Therefore, it is evident that analysis using pERK as a biochemical marker is useful for the characterization of spinal pain transmission.

It should be noted that the CNQX-insensitive but NMDA or NK1 receptor antagonists-sensitive spinal pain transmission was observed in the present study, since many electrophysiological studies show predominant contribution of AMPA/kainate receptor in the generation of EPSC in spinal neurons [17,18]. In the study by Miller and Woolf [17], CNQX reduced the fast component (Aβ) of EPSC to ~30%, while did the slow component (Aδ +C) only to ~50%. As NMDA receptor antagonist inhibited the slow (Aδ +C), but not fast (Aβ) component of EPSC, it is suggested that there exists the CNQX-insensitive NMDA-receptor-mediated slow component (Aδ +C) in the pain transmission. This view is further supported by the finding that the addition of NMDA elicited an inward current at holding potentials of -60 mV in the presence of Mg^2+ ^[19]. Although the machinery underlying CNQX-insensitive NMDA current remains to be determined, there is a possibility for the predominant NMDA receptor activation. Takasu et al. [20] reported that CNQX-insensitive NMDA receptor-mediated Ca^2+ ^influx in the presence of ephrinB2-EphB2 activation, which activates NMDA receptor through a Src-mediated phosphorylation. In nerve-injured mice, the EPW thresholds in response to 2000 and 250 Hz stimuli were significantly decreased (hypersensitized), while the threshold in response to a 5 Hz stimulus was increased (hyposensitized). These results are also consistent with our previous study using the algogenic-induced paw flexion (APF) test, showing that prostaglandin I_2 _(PGI_2_) agonist-induced A-fiber responses were hypersensitization and that substance P-induced C-fiber responses were diminished in nerve-injured mice [21-23]. It should be noted that the 2000 Hz stimuli-induced hypersensitization in injured mice was completely abolished by intrathecal administration of NMDA receptor antagonists, but not by CNQX. This fact suggests that non-nociceptive information transmitted through Aβ-fibers via AMPA/kainate receptor-mediated spinal neurotransmission is converted into NMDA receptor-sensitive nociceptive information in nerve-injured mice. By contrast, Aδ-fiber-mediated nociceptive responses were also hypersensitized, but the spinal antagonism remained unchanged.

Consistent with the C-fiber hypoalgesia and the Aδ-fiber hyperalgesia observed in the EPW test, the numbers of pERK-positive neurons throughout C-fibers and Aδ-fibers were decreased and increased, respectively, in nerve-injured mice. It is interesting that pERK-signals in smaller DRG neurons (<18 μm) were selectively lost following nerve injury, but the underlying machineries remain to be determined. By contrast, the number of Aδ-fiber stimuli-induced pERK-signals in spinal neurons was increased in injured mice, although no significant change was observed in DRG neurons. The selective activation of spinal neurons in terms of pERK-signals may be explained by the up-regulation of the voltage-dependent calcium channel α_2_δ-1 (Caα_2_δ-1) subunit [24,25], which enhances spinal neurotransmission, leading to an activation of post-synaptic neurons.

The important finding in this study is that Aβ-fiber-induced ERK activation, which is not observed in the naïve state, was detected in the neuropathic pain state, significantly, in the superficial laminae of the dorsal horn, which normally receive nociceptive neural projections. The results obtained using transcutaneous Aβ-fiber stimulation by the Neurometer^® ^are highly consistent with previous studies using low-threshold electrical stimulation to the sciatic nerves of nerve-injured rats [26,27]. In the present study, we succeeded in the pharmacological characterization of Aβ-fiber-induced ERK activation caused by nerve injury. The Aβ-fiber-induced pERK activation in spinal neurons of nerve-injured mice was blocked by NMDA receptor antagonists (AP-5 and MK-801), but not by an AMPA/kainate receptor antagonist (CNQX), consistent with the present findings that Aβ-fiber hypersensitization was blocked by NMDA antagonists in the EPW test. These results strongly suggest that the Aβ-fiber stimulation may activate spinal neurons that were originally innervated by nociceptive C-fibers or Aδ-fibers. As nerve injury causes hyposensitivity to C-fiber stimulation, the interaction between Aβ-fibers and Aδ-fibers seems to be more important for the neural plasticity observed in the neuropathic pain state. There is an alternative possibility that Aβ-fibers become hypersensitive through an alteration of gene expression, but such a mechanism can not explain the plasticity, since the present study showed no significant ERK activation in laminae III–V, which are expected to be innervated by Aβ-fibers in naïve animals [28].

Regarding the mechanisms underlying the neural plasticity in neuropathic pain, there are reports that ephaptic discharges, that is, abnormal neural sensitization in pre-synaptic neurons or neural sprouting in the spinal dorsal horn, occurs [29-31]. From the recent finding that demyelination of A-fibers and allodynia occur through mechanisms associated with the lysophosphatidic acid receptor in the dorsal roots of nerve-injured mice [25], we have proposed that demyelination-induced loss of insulation may cause abnormal cross-talk among A-fibers and sprouting, resulting in a functional switch of innocuous stimulus to painful perception [8,23].

## Conclusion

The present study demonstrates that nerve injury causes a pharmacological switch from AMPA/kainate receptor to NMDA receptor neurotransmission through Aβ-fibers. The present findings may provide the molecular mechanisms underlying the clinical findings that systemic and intrathecal treatments with NMDA-receptor antagonists cure neuropathic allodynia and hyperalgesia [32,33].

## Methods

### Animals

Male ddY mice weighing 20–24 g were used after adaptation to laboratory conditions: 22 ± 2°C, 55 ± 5% relative humidity and a 12-hour light/dark cycle with food and water ad libitum. All procedures were approved by the Nagasaki University Animal Care Committee and complied with the recommendations of IASP [34].

### Drugs and treatment

The following drugs were used: D-2-amino-5-phosphonovaleric acid (AP-5) (Tocris Cookson, USA), (+)-5-methyl-10,11-dihydro-5H-dibenzo [a,d]cyclohepten-5,10-imine hydrogen maleate (MK-801) (Sigma, USA) and 6-cyano-7-nitroquinoxaline-2,3-dione (CNQX) (Research Biochemicals International, USA). CP-99994 was generously provided by Pfizer Pharmaceuticals (Sandwich, Kent, UK). All drugs except CNQX were dissolved in artificial cerebrospinal fluid (aCSF) comprising 125 mM NaCl, 3.8 mM KCl, 2.0 mM CaCl_2_, 1.0 mM MgCl_2_, 1.2 mM KH_2_PO_4_, 26 mM NaHCO_3 _and 10 mM D-glucose (pH 7.4). CNQX was initially dissolved in DMSO at 300 nmol/5 μL and further diluted in aCSF for intrathecal injection. As a control (vehicle), DMSO was diluted with aCSF in the same proportion as in the CNQX solution. All drugs were given intrathecally in a volume of 5 μl, 10 min before electrical stimulation.

### Partial ligation of sciatic nerve

Partial ligation of the sciatic nerves of mice was performed under pentobarbital (50 mg/kg, i.p.) anesthesia, following the method of Malmberg and Basbaum [10]. Briefly, the common sciatic nerve of the right hind limb was exposed at high thigh level through a small incision and the dorsal half of the nerve thickness was tightly ligated with a silk suture. A sham-operation was performed similarly except without touching the sciatic nerve. The experiments in the present study were carried out 7 days after nerve ligation or sham-operation, as maximum thermal hyperalgesia and mechanical allodynia were observed at this time-point [22,25].

### Electrical stimulation-induced paw withdrawal (EPW) test

The EPW test was performed as described previously [35]. Briefly, electrodes (Neurotron Inc., Baltimore, MD) were fastened to the right plantar surfaces and the insteps of mice. Transcutaneous nerve stimuli (sine-wave pulses of 2000, 250 or 5 Hz) were applied using a Neurometer CPT/C (Neurotron Inc.). The minimum intensity (μA) at which each mouse withdrew its paw was defined as the current threshold. All behavioral experiments were carried out by investigators blinded to the drug treatment.

### Stimulation for ERK phosphorylation experiments

For the spinal antagonism experiment, sham-operated or nerve-injured mice were intrathecally treated with drugs or aCSF, and deeply anesthetized with sodium pentobarbital (50 mg/kg, i.p). The electrodes were fastened with tape to the operated right plantar surface and instep. After 10 min, nerve stimuli (2000, 250 and 5 Hz) were applied. The current intensities of the 2000, 250 and 5 Hz stimuli were 1000 μA, 2000 μA and 2000 μA, respectively, and the duration of stimulation was 1 min. As ERK activation requires relatively high-intensity noxious stimulation [3,4], we adopted thresholds that were higher than the behavioral withdrawal threshold determined in the EPW test. Control treatment was performed similarly, except without electrical stimulation. Two min after electrical stimulation, mice were immediately perfused with ice-cold PBS, followed by cold 4% paraformaldehyde solution. The L4-5 spinal cord and DRGs were isolated, postfixed for 3 hours, and cryoprotected overnight in 25% sucrose solution. The tissues were fast-frozen in cryo-embedding compound on a mixture of ethanol and dry ice and stored at -80°C until use. The ventral horn contralateral to the stimulation was marked with a small cut so that the ipsilateral side of spinal cord could be identified after sectioning. DRGs were cut on a cryostat at a thickness of 10 μm, thaw-mounted on silane-coated glass slides, and air-dried overnight at room temperature (RT). Spinal cords were cut on a cryostat at a thickness of 30 μm, collected in PBS solution containing 0.1% sodium azide, and processed as free-floating sections.

### Immunostaining for phosphorylated ERK (pERK)

To perform DAB immunostaining for pERK, slide-mounted DRG sections and free-floating spinal cord sections were incubated in 1% H_2_O_2 _for 30 min and washed with TBS. The sections were incubated with blocking buffer containing 3% BSA in TBST (0.1% Triton X-100 in TBS) and subsequently reacted overnight at 4°C with anti-pERK antibodies (anti-phospho-p44/42 MAP kinase antibodies, 1:500; Cell Signaling Technology, MA) in blocking buffer. After thorough washing, the sections were incubated with biotinylated anti-rabbit IgG secondary antibody (1:500; Vector, CA) for 60 min at RT, and subsequently with ABC complex (Vector, CA) at RT for 60 min. The pERK immunoreactivities were visualized by incubation with a solution containing 0.02% 3,3'-diaminobenzidine tetrahydrochloride (DAB; Dojindo, Japan), 0.0051% H_2_O_2 _in 0.05 M Tris-HCl buffer (pH 7.6) until brown reaction products appeared. The reaction was stopped by washing with TBS. Sections were dehydrated through a series of ethanol solutions, cleaned in xylene, and coverslipped. For double immunostaining, we used the following antibodies: a mouse monoclonal antibody against neuron-specific nuclear protein (anti-NeuN, 1:500; Chemicon, CA), Alexa Fluor 594-conjugated anti-mouse IgG and Alexa Fluor 488-conjugated anti-rabbit IgG (1:300; Molecular Probes, CA). All sections were evaluated by microscopy (Keyence, Tokyo, Japan).

Measurements of the number of pERK-positive cells in the L4-5 spinal dorsal horn and the diameters of pERK-positive DRG cells were carried out using BZ Image Measurement software (Keyence, Tokyo, Japan). To assess the immunoreactivity of spinal dorsal horn neurons, we counted only those immunoreactive cells with an S/N ratio of 3.0 or more and a diameter of >5 μm. For the background activity, we assessed the intensities in the gracile fasciculus regions of white matter. The quantification of positive cells was performed using 5–6 sections per mouse. To assess the total number of DRG cells and the diameters of pERK-positive cells, considering the biases introduced by the stereological approach, we used only those cells stained by Methylgreen pyronin using adjacent sections. Quantification of positive cells was performed using 5–6 sections per mouse.

### Statistical analysis

Differences between multiple groups were analyzed using a one-way ANOVA and a Tukey-Kramer multiple comparison post-hoc analysis. Changes in the number of pERK-positive cells, between sham-operated and nerve-injured mice, were analyzed using an unpaired Student's *t*-test. The criterion of significance was set at *p *< 0.05. All results are expressed as means ± S.E.M.

## List of abbreviations

EPW: electrical stimulation-induced paw withdrawal; EPF: electrical stimulation-induced paw flexion; DRG: dorsal root ganglion; ERK: extracellular signal-regulated kinase; pERK: phosphorylated extracellular signal-regulated kinase; aCSF: artificial cerebrospinal fluid.

## Competing interests

The authors declare that they have no competing interests.

## Authors' contributions

MM is responsible for experimental design, immunohistochemical experiments, and writing the manuscript. LM participated in behavioral experiments. WX participated in immunohistochemical experiments. All authors read and approved the final manuscript.
